# Cognitive function and the agreement between self-reported and accelerometer-accessed physical activity

**DOI:** 10.1186/s12877-018-0747-x

**Published:** 2018-02-21

**Authors:** Florian Herbolsheimer, Matthias W. Riepe, Richard Peter

**Affiliations:** 1 Institute of the History, Philosophy and Ethics of Medicine, Ulm University, Parkstraße 11, 89073 Ulm, Germany; 2 Division of Mental Health & Old Age Psychiatry, Psychiatry II, Ulm University, Günzburg, Germany

**Keywords:** Accelerometer, Physical activity questionnaire, Physical activity, Cognitive function

## Abstract

**Background:**

Numerous studies have reported weak or moderate correlations between self-reported and accelerometer-assessed physical activity. One explanation is that self-reported physical activity might be biased by demographic, cognitive or other factors. Cognitive function is one factor that could be associated with either overreporting or underreporting of daily physical activity. Difficulties in remembering past physical activities might result in recall bias. Thus, the current study examines whether the cognitive function is associated with differences between self-reported and accelerometer-assessed physical activity.

**Methods:**

Cross-sectional data from the population-based Activity and Function in the Elderly in Ulm study (ActiFE) were used. A total of 1172 community-dwelling older adults (aged 65–90 years) wore a uniaxial accelerometer (activPAL unit) for a week. Additionally, self-reported physical activity was assessed using the LASA Physical Activity Questionnaire (LAPAQ). Cognitive function was measured with four items (immediate memory, delayed memory, recognition memory, and semantic fluency) from the Consortium to Establish a Registry for Alzheimer’s Disease Total Score (CERAD-TS).

**Results:**

Mean differences of self-reported and accelerometer-assessed physical activity (MPA) were associated with cognitive function in men (r_s_ = −.12, *p* = .002) but not in women. Sex-stratified multiple linear regression analyses showed that MPA declined with high cognitive function in men (β = −.13; *p* = .015).

**Conclusion:**

Results suggest that self-reported physical activity should be interpreted with caution in older populations, as cognitive function was one factor that explained the differences between objective and subjective physical activity measurements.

**Electronic supplementary material:**

The online version of this article (10.1186/s12877-018-0747-x) contains supplementary material, which is available to authorized users.

## Background

Physical inactivity is an important health behavior, while it has been proposed that reduced levels of physical activity are a risk factor for obesity, cardiovascular diseases, dementia and other chronic conditions [1, 2]. In order to identify the association between physical activity and these health outcomes, epidemiological studies have often used self-reported physical activity measurements.

In the past, most studies have relied on recall processes to obtain information about physical activity with recall frames ranging from one week to a lifetime. To date, research on physical activity has started to validate self-reports by using objectively-assessed instruments, which, however, mostly have resulted in moderate correlations [3]. Pedometers and self-reported physical activity correlated only moderately r = .3 [4] and correlations between accelerometer and questionnaires mostly ranged from r = .3 to r = .5 (e.g., [5–7]). It is therefore important to determine the factors that explain the observed deviations between different physical activity measurements. Literature reviews including large epidemiological studies have documented that self-reported physical activity tended to be overreported when comparing it to accelerometer-assessed physical activity [6, 8, 9].

Cognitive function might be one factor that explains deviations between self-reported and accelerometer-assessed physical activity – particularly among older adults. Cognitive limitations are more prevalent in older populations, since recalling behavior is a complex cognitive task [10, 11]. Cumming & Klineberg [12] found that cognitively impaired older adults reproduced less accurate long-term physical activity recollections. These results were based on a comparison between physical activity questionnaires and physical activity diaries. Memory processes were hypothesized to influence self-reported physical activity. Durante & Ainsworth [13] identified from a cognitive psychological perspective different steps of information retrieval that could lead to invalid data. In order to report past physical activity correctly, participants had to recall 1) the types of activities they have done, 2) the frequencies of the activity and 3) the date of the activity [13]. In addition, every recall step in the recollection might become more difficult the longer the period and the more distant the reference periods were.

Demographic characteristics were also identified in the literature to influence the accuracy of the two physical activity assessments. Higher correlations were found in highly educated [14–16] and normal weight individuals [17, 18]. A further finding that is seemingly consistent across the literature is the fact that self-reported and objectively-assessed physical activity is more strongly associated in men in comparison to women [9, 19, 20]. This might be because older women’s physical activity substantially differs in terms of physical activity levels, types and preferred locations [21]. Women also engaged in more light physical activity, which is the most difficult type of physical activity to recall [22]. That makes it important to separately analyze physical activity in men and women. The same applies to older age groups because they engaged mostly in light to moderate intensity activities which explains a weaker correlation among older adults compared to younger age groups [5, 9].

Another explanation of the moderate agreement might also be found in the fact that physical activity questionnaires use ambiguous terms like “moderate intensity” or “leisure time” and instructions are sometimes difficult to understand. This might lead to difficulties with comprehending instructions for researchers and respondents [23, 24]. Furthermore, response behavior might follow socially desirable expectations that might lead to diverging results in physical activity responses [25].

With this in mind, the study aimed to: 1) Compare self-reported physical activity with accelerometer-assessed physical activity, 2) examine if both measures were stronger correlated in men, 3) and investigate the role of cognitive function in explaining disagreements between self-reported and accelerometer-assessed physical activity.

Based on the aforementioned literature, the difference between both physical activity measurements is expected to be associated with cognitive function. We expect both physical activity measurements to be more strongly associated in individuals with high cognitive function in comparison to older adults with low cognitive function.

## Methods

### Study population

The ActiFE study recruited community-dwelling older adults from the greater area of Ulm, Germany. Participants aged between 65 and 90 were randomly selected by local statistical offices. Inclusion criteria for the study were as follows: Participants were required (i) not to be institutionalized, (ii) to be German-speaking, and (iii) not to use a wheelchair. Furthermore, cognitively impaired or demented older adults (≤ 25 on the Mini-Mental State Examination (MMSE)) were excluded from the following analyses. A stratified sample has been drawn from three age groups (65–69; 70–79; 80–90) over-representing the oldest old [26]. In total, 1506 eligible older adults agreed to participate (participation rate 19.8%). The accelerometer measurement and the interviews took place between April 2009 and June 2010. Interviews in 188 cases could not be analyzed due to missing physical activity data. More particularly, in these cases, there were 43 missing questionnaire data and 145 missing accelerometer values. 128 individuals were classified as cognitively impaired, 12 cases were dropped due to insufficient information about cognitive function and in six cases, too much time (< 11 days) passed between the two measurements (accelerometer and questionnaire). The final study sample consisted of 1172 persons.

### Measures

#### Accelerometer-assessed physical activity

A uni-axial accelerometer (activPAL, PAL technologies ltd., Glasgow, UK) assessed daily walking duration [27]. The activPAL™ represents a single-axis accelerometer that is based on posture detection in combination with vertical acceleration and samples body accelerations at 10 Hz (10 times per second). The activPAL™ generates three forms of activity data: Walking, quiet standing and sitting/lying. In previous studies, the activPAL™ has been demonstrated to be highly accurate [28] and shows high inter-device reliability [27]. The present analyses focused on walking time. Participants were asked to wear the monitor that was attached to the leg for a period of 7 days. Accelerometer data were excluded from further analysis if they recorded less than 24 h a day. Average physical activity time was calculated as the total walking duration (6 * mean of weekday and Saturday + 1* Sunday) divided by seven and was expressed as minutes per day.

### Self-reported physical activity

The Longitudinal Aging Study Amsterdam Physical Activity Questionnaire (LAPAQ) was applied to assess self-reported physical activity. The LAPAQ was found to be highly correlated with a diary covering 7 days (*r* = .68, *p* < .001) and moderately correlated with a pedometer (*r* = .56, *p* < .001) [29]. The LAPAQ asks for the frequency (i.e., How many times did you ….. during the past 2 weeks?) and duration (i.e., How long did you usually …. each time?) for six activities in the previous two weeks. The activities were daily walking, daily cycling, gardening, light household work, heavy household work, and a maximum of 2 types of sports. Daily walking and daily cycling were not classified as sports if they were meant to perform everyday activities, like walking or cycling to the supermarket. In order to calculate the average daily activity, the frequency and duration were multiplied and divided by 14 days. A modified total activity score was calculated by adding up three of the original six physical activities, which were: walking, cycling, and sports (LAPAQ-M). Light and heavy household activities were excluded from the calculation because a factor analysis (not shown) revealed a two-factorial solution showing that household activities correlated with a different factor than the other activities. Furthermore, it is questionable whether household activities provide all of the benefits that are normally associated with meeting the physical activity guidelines [30]. Gardening was excluded from the index, as additional analyses revealed a better agreement between self-reported and accelerometer-assessed physical activity without this item. Extreme outliers (> 5 standard deviation (SD); (*n* = 8 to *n* = 14)) of each single activity were set to the value of the 5th SD.

### Cognitive function

All participants underwent a neuropsychological test battery established from the Consortium to Establish a Registry for Alzheimer’s Disease (CERAD) [31]. The CERAD has been found to be a valid and reliable measurement of cognitive function in a normal aging population, in older adults with mild cognitive impairment, and in persons with Alzheimer disease [32]. The present study used the CERAD Total Score (CERAD-TS) [32] that included measurements of 1) immediate memory (ten words, three trials), 2) delayed memory (delayed recollection of the ten words), 3) recognition memory (recognition of the ten words out of twenty) and 4) semantic fluency (animals). A higher score on each subtest indicates better cognitive function. In accordance to Chandler and colleagues (2005), a cap of 24 was also placed on the verbal fluency item and the MMSE was excluded because of its global nature. The “Boston naming test” and “constructional praxis” were not assessed in the current study [32]. Following Chandler (2005) we calculated a raw total score (Cronbach’s α = .71) representing an index of global neurocognitive functioning ranging from 0 to 74 with higher scores indicating better cognitive functioning. Other methods like constructing a total score such as a *z* – score transformation that put equal weights on each subtest were explored and revealed same results.

### Covariates

Other studies have identified several confounders that substantially influenced the association between accelerometer-assessed and self-reported physical activity [15]. To address this issue, the variables of sex, age, self-rated health, an indicator variable for the interviewer, average temperature, and body mass index (BMI) were considered as potential confounders. Self-rated health was measured with one item from the 12-item Short Form Health Survey: “In general would you say your health is” including poor, fair, good, very good, and excellent [33]. All five interviewers were considered as dichotomous variables in order to adjust for possible interviewer effects. A local weather station provided the maximum temperature (°C) during the measurement period of the accelerometer. Furthermore, sex and age (in years) were assessed.

### Missing values

Complete data were provided by 77.8% of all participants. The imputation procedure was exclusively applied if one out of four cognitive function items was missing (*n* = 57). Previous research has shown that physical activity patterns significantly varied on Sundays [34]. Therefore, accelerometer-assessed physical activity on Sundays was imputated (regression estimate) and the total accelerometer physical activity score was adjusted if Sunday was missing (*n* = 55). The results were nearly identical in supplementary analyses using list-wise deletion. However, the analytic sample includes the imputed data because it reduces concerns about sample size and the potential biases imposed by dropping cases with item-specific missing data.

### Statistical analysis

The differences in characteristics between older men and women were examined using independent t-tests for continuous data and chi-square tests for categorical data. For skewed continuous variables, the differences between men and women were tested using a Mann-Whitney-U test. In order to describe the relationship between self-reported and accelerometer-assessed physical activity, Spearman’s rho and a Bland-Altman plot were calculated.

The mean difference of self-reported and accelerometer-assessed physical activity (MPA) (self-reported minus accelerometer-assessed) was taken as the outcome in linear regression models. First, we tested sex as moderator by examining the interaction effect between sex and cognitive function on MPA. If the interaction term was significant, sex-specific associations between cognitive function and MPA were calculated [35]. The models were adjusted step by step, first reporting the bivariate associations between cognitive function and MPA. In a second step, we adjusted for all aforementioned confounding variables: Age, BMI, average temperature, self-rated health, and for interviewer effect using an indicator variable for the interviewer. The data were analyzed using STATA software, version 10.1 (StataCorp LP, College Station, TX).

## Results

Table 1 presents the characteristics of the study population. The majority of respondents were male (56%) and had received less than a college education. The mean age of 75.3 years (*SD* = 6.5; range = 65–90) indicates that the study population represents primarily an older age group. A mean daily physical activity time of 104.7 min (*SD* = 39.9) was detected by the activPAL™. Participants had a mean of 5.7 (*SD* = 0.9) valid (24 h) days of accelerometer wear. A mean of 96.1 min (*SD* = 79.2) of daily physical activity was calculated based on self-reported information derived from the LAPAQ-M and representing the sum of walking, cycling, and sports. Women were found to report less physical activity, showed lower education levels and had higher levels of cognitive function. Both sexes were found to be equally active according to accelerometer-assessed physical activity.

**Table 1:** Sample characteristics of respondents

	Mean (*SD*) / Percentage						*p*-value
	Total		Men		Women		
	(*n* = 1172)		(*n* = 658)		(*n* = 514)		
Physical activity							
Self-reported (minutes / day)	96.1	(79.2)	101.6	(82.3)	89.1	(74.8)	.008
Accelerometer-assessed (minutes / day)	104.7	(39.9)	105.1	(40.9)	104.1	(38.5)	.679
Mean differences^a^ (minutes / day)	−8.5	(75.2)	−3.5	(77.0)	−15.0	(72.6)	.002
Memory							
Cognitive function^b^ (0–74)	53.1	(9.3)	51.6	(9.2)	55.0	(9.1)	< .001
MMSE^c^ (0–30)	28.5	(1.2)	28.5	(1.2)	28.6	(1.2)	.363
Word list learning (0–30)	18.7	(3.9)	18.0	(3.7)	19.6	(3.9)	< .001
Word list recall (0–10)	5.6	(2.3)	5.2	(2.3)	6.0	(2.3)	< .001
Word list recognition discriminability (0–10)	8.7	(1.8)	8.4	(1.9)	8.9	(1.6)	< .001
Verbal fluency (0–24)	20.2	(4.2)	20.0	(4.3)	20.4	(3.9)	.132
Control Variables							
Age (years)	75.3	(6.5)	75.7	(6.5)	74.8	(6.6)	.011
School education (%)							
low (<=9 years)	55.3		53.0		58.0		< .001
middle (10 years)	23.3		19.3		28.6		
high (> 10 years)	21.4		27.7		13.4		
Body mass index (kg/m^2^)^d^	27.6	(4.0)	27.9	(3.5)	27.2	(4.5)	.002
Self-rated health^e^	3.1	(0.7)	3.1	(0.7)	3.1	(0.7)	.984
Average temperature (°C)	12.2	(9.3)	12.2	(9.5)	12.2	(9.1)	.749

Notes. ^a^questionnaire - accelerometer; ^b^based on 4 items (immediate memory, delayed memory, recognition memory and semantic fluency); ^c^Mini Mental State Examination; ^d^weight/height^2^; ^e^ranging from 1 to 5, higher values indicate better health

**Fig. 1:**
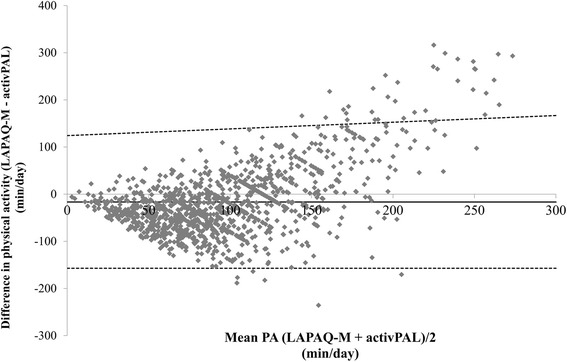
Bland-Altman plot of minutes per day for physical activity from the activPAL™ and the LAPAQ-M. The mean error scores are illustrated by a solid horizontal line and the limits of agreement (+ − 1.96 SD from the mean) are shown as dashed horizontal lines

Self-reported and accelerometer-assessed physical activity were moderately correlated (r_s_ = .41) with higher Spearman correlations for men (r_s_ = .43) than for women (r_s_ = .39). The Bland-Altman plot (Fig. 1) illustrates the agreement between the accelerometer and self-reported physical activity. The differences between the two measurements increased with higher levels of physical activity.

Analyzing each memory task separately (i.e., immediate memory, delayed memory, recognition memory, and semantic fluency), we found the strongest correlation between MPA and recognition memory (*r*_s_ = −.10; *p* < .001), followed by immediate memory, delayed memory, and a poor association with semantic fluency. The association was stronger in older men than in older women.

**Fig. 2:**
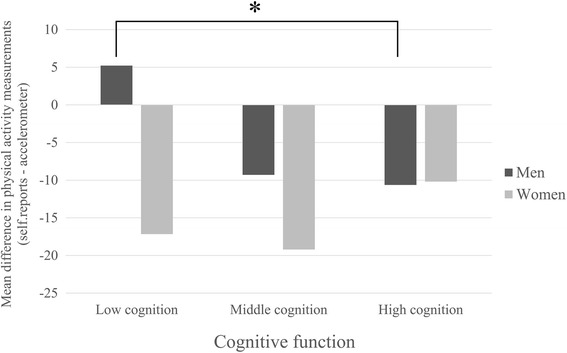
Mean physical activity difference in older adults, stratified by tertiles of cognitive function and sex. Mean values and significances are presented

Figure 2 shows the association between cognitive function and MPA. Here, higher values indicated more self-reported physical activity whereas lower values point to more accelerometer-assessed physical activity. MPA increased with lower cognitive function in men. MPA significantly differed in men between the lowest cognitive function tertile in comparison to the highest cognitive function tertile (*p* < .05). In contrast, MPA was almost stable across cognitive function tertiles in women.

Accordingly, older women scored significantly lower in multivariate analyses, and sex significantly moderated the association between MPA and cognitive function (β = .10; *p* = .008). Consequently, all subsequent models were separately calculated for men and women. In multiple regression analyses, we investigated the role of the cognitive function in explaining MPA (self-reported minus accelerometer-assessed physical activity) (Table 2). The first analysis showed that cognitive function had no effect on MPA in women. In men, we observed a negative bivariate association between cognitive function and MPA (β = −.09; *p* = .016). Accelerometer-assessed physical activity also served as a predictor. This adjustment was made in order to account for the fact that higher disagreements between the two physical activity measurements have been repeatedly found in highly physically active individuals. In men, the bivariate association remained after adjusting for confounding variables in model 2 (β = −.13; *p* = .015). The higher the cognitive function, the lower the MPA levels in men.

**Table 2:** Cross-sectional associations between MPA (mean differences of self-reported and accelerometer-assessed physical activity) and cognitive function, stratified by sex

	Men (*n* = 658)		Women (*n* = 514)	
	β	*p*-value	β	*p*-value
Model 1	−.09	.016	.06	.151
Model 2	−.13	.015	.08	.079

Note. β = standardized beta coefficient; cognitive function is based on an index of 4 items (immediate memory, delayed memory, recognition memory and semantic fluency)

Model 1: Bivariate association between mean differences of self-reported and accelerometer-assessed physical activity and cognitive function

Model 2: Model 1 additionally adjusted for age, body mass index, physical activity (accelerometer-assessed), interviewer (as cluster variable), self-rated health, and the average temperature

In summary, older men with low cognitive function reported more physical activity compared to accelerometer-assessed physical activity.

## Discussion

The current study revealed that cognitive function was significantly associated with differences between self-reported and accelerometer-assessed physical activity. Older men with low cognitive function reported proportionally more physical activity in relation to accelerometer-assessed physical activity than individuals with high cognitive function did. Differences between accelerometer-assessed and self-reported physical activity might be due to difficulties in comprehending a physical activity questionnaire or the inability to correctly recall past physical activity behavior. The results were robust, as they did not change after including a variety of confounding variables, such as age, interviewer, and body-mass-index. These findings suggest that cognitive function is an important factor for comparing self-reported and accelerometer-assessed physical activity.

To our knowledge, there is no study that has investigated the relationship between cognitive function and discrepancies in physical activity measurements. Yet, past studies have reported findings regarding the impact of educational differences on the validity of physical activity measurements. Educational attainment and cognitive function could be considered as related measurements because older adults with a middle or high level of education performed better on cognitive tests [36]. The effect of education pointed in the same direction as our results. The level of agreement between self-reported and accelerometer-assessed physical activity increased with higher levels of education [16, 37]. More over-reporting of physical activity was found in older adults with lower than in medium and higher levels of education [14, 15].

Overall, men reported more physical activity than women, although there was no difference in accelerometer-assessed physical activity, which is in accordance with a previous study [5]. Differences in self-reports might come from sex-specific physical activity behavior. Hagstromer and colleagues [38] have suggested that men in general engage in physical activities that might be inefficiently recorded using an accelerometer. Older men and women have been found to engage in different kinds of activities in relation to the intensity, frequency and the location of physical activity [21] with varying difficulty in recollecting such activities. Additionally, the self-reported physical activity might also be biased by socially desirable response behavior [10] because the benefits of physical activity are well known in the population and respondents were aware that the current study focused on physical activity. The Bland-Altman plot (Fig. 1) found that physically active older adults overreported their physical activity level when it was compared to accelerometer values. The differences between the two measurements increased with higher overall physical activity levels. This was in agreement with prior studies that compared accelerometers to self-reports [5, 19, 20, 38]. These results indicated that either older adults with high physical activity levels overreported physical activity or the accelerometer is less suitable to capture high physical activity levels.

In our study, both physical activity measurements were moderately correlated in men and women (*r*_*s*_ = .41), which is in accordance with previous studies. These studies usually revealed coefficients correlated between .23 and .46 when examining the agreement between accelerometer-assessed and self-reported physical activity [39]. Thus, a majority of physical activity questionnaires had acceptable reliability and moderate validity at best. The same relations held for newly developed physical activity questionnaires which do not appear to perform substantially better than the existing ones in terms of reliability and validity [23]. Questionnaires and accelerometers might capture different aspects of physical activity and might be biased to over- or underestimate physical activity in certain groups. Neither the questionnaire nor the accelerometer can be taken as a gold standard, but rather each measurement contains components of random and systematic errors. Poor agreements between both measurements raise concerns about the conclusions that have been drawn depending on self-reported physical activity and various health outcomes. Winkler and colleagues [40] reported that the measurement error between self-reports and accelerometery might even appear to be affected by the intervention per se. There is consequently a need to identify factors that explain the disagreement between self-reported and accelerometer-assessed physical activity.

The discrepancy between self-reports and accelerometery might become even more problematic when accurate population-based physical activity levels are required for public health offices in order to explore physical activity trends and evaluate if certain initiatives have been able to target the studied population. Depending on the measurement technique, population-based prevalence of physical inactivity strongly differed [41]. Additionally, physical activity interventions have been found to be more effective if they are based on objective measurements instead of self-reports [42]. Because measurement error cannot be definitively attributed to questionnaires or accelerometers, it would be prudent to measure both in future studies [40].

This study has several strengths, including its large sample, the use of a fixed attached accelerometer that recorded 24 h a day, and the use of the CERAD Total Score, which is a comprehensive instrument to assess the domains of cognitive function. Our study also has some limitations. First, the final models only showed rather small effect sizes. This might be a result of using multivariate models that adjusted for confounders with a person’s physical activity level as the most important one. Second, although the study population included in the final analysis did not significantly differ in terms of age and sex from persons who were excluded from the following analyses and the sample covered was randomly selected, we observed that participants tended to be younger in comparison with non-respondents. Furthermore, women were underrepresented in the study and significantly scored higher on cognitive function compared to men (see Additional file 1: Figure S1, which illustrates the distribution of cognitive function stratified by sex). This limited the variability of the cognitive function measurement, suggests the presence of a selection bias and that a part of the observed sex difference could possibly be traced to differences in cognitive function. Third, the comparison with other studies might be limited since different instruments of self-reported physical activity (IPAQ, LAPAQ) and accelerometer-assessed physical activity (Actigraph, activPAL™) were used. Fourth, the time intervals between self-reported and accelerometer-assessed physical activity assessments did not perfectly match. The time intervals slightly deviated because self-reported physical activity referred to the previous 14 days, whereas the accelerometer measures only up to 7 days. However, physical activity can be regarded as a constant and routine behavior with small intra-individual variation. An investigation of the day-to-day variability of time that is spent walking advises that one to three days are sufficient to describe physical activity patterns over a week [43]. Additional sensitivity analyses also showed similar results comparing persons with varying overlapping periods. Finally, we are aware that physical activity questionnaires might capture only a selection of activities, whereas the accelerometer assessed every lower body movement. However, this does not explain why cognitive function was associated with systematic variation in self-reported physical activity in relation to the accelerometer-assessed physical activity.

## Conclusion

To the best of our knowledge, this was the first study of its kind in a population of older adults that took cognitive function to be an independent factor in describing discrepancies in subjective and objective physical activity measurements. Because physical activity is regarded as a key to successful aging, it needs to be better understood how self-reported and accelerometer-assessed physical activity differ. Future studies need to identify aspects of the physical activity that are most critical for health in older adults and pay closer attention to measurement issues, since the effect of physical activity might be under - or overestimated in certain groups if it is derived exclusively from questionnaires.

## Additional file

Additional file 1: **Figure S1.** Figure that illustrates the distribution of cognitive function in both sexes. Ttif (TIFF 3086 kb)

## Abbreviations

ActiFE: Activity and Function in the Elderly
BMI: Body Mass Index
CERAD –TS: Consortium to Establish a Registry for Alzheimer’s Disease – Total Score
IPAQ: International Physical Activity Questionnaire
LAPAQ: LASA Physical Activity Questionnaire
LASA: Longitudinal Aging Study Amsterdam
MMSE: Mini Mental State Examination
MPA: Mean difference of self-reported and accelerometer-assessed physical activity
PA: Physical activity
SD: Standard deviation
